# Protective Role of L-3-*n*-Butylphthalide in Cognitive Function and Dysthymic Disorders in Mouse With Chronic Epilepsy

**DOI:** 10.3389/fphar.2018.00734

**Published:** 2018-07-11

**Authors:** Xiaowen Ye, Zhouyi Rong, Yanfang Li, Xintian Wang, Baoying Cheng, Yiyun Cheng, Haijuan Luo, Yue Ti, Xiaohua Huang, Zhaoji Liu, Yun-wu Zhang, Weihong Zheng, Honghua Zheng

**Affiliations:** ^1^Department of Neurology, Affiliated Zhongshan Hospital, Xiamen University, Xiamen, China; ^2^Fujian Provincial Key Laboratory of Neurodegenerative Disease and Aging Research, Institute of Neuroscience, College of Medicine, Xiamen University, Xiamen, China; ^3^Department of Neurology, Affiliated Zhongshan Hospital, School of Clinical Medicine, Fujian Medical University, Xiamen, China; ^4^Department of Neurology, Affiliated Zhongshan Hospital, Graduate School of Fujian University of Traditional Chinese Medicine, Xiamen, China; ^5^Basic Medical Sciences, College of Medicine, Xiamen University, Xiamen, China; ^6^Shenzhen Research Institute, Xiamen University, Shenzhen, China

**Keywords:** l-3-*n*-butylphthalide, chronic epilepsy, learning and memory, anxiety and depression, protection

## Abstract

Epilepsy is a common neurological disease with recurrent seizures and neurobehavioral comorbidities, including cognitive impairment and psychiatric disorders. Recent studies suggest that L-3-*n*-butylphthalide (NBP), an extract from the seeds of *Apium graveolens* Linn. (Chinese celery), ameliorates cognitive dysfunction in ischemia and/or Alzheimer’s disease animal models. However, little is known about the role of NBP in epilepsy and the associated comorbidities. Here, using a pilocarpine-induced chronic epileptic mouse model, we found that NBP supplement not only alleviated seizure severity and abnormal electroencephalogram, but also rescued cognitive and emotional impairments in these epileptic mice. The possible underlying mechanisms may be associated with the protective role of NBP in reducing neuronal loss and in restoring the expression of neural synaptic proteins such as postsynaptic density protein 95 (PSD95) and glutamic acid decarboxylase 65/67 (GAD65/67). In addition, NBP treatment increased the transcription of neuroprotective factors, brain-derived neurotrophic factor and Klotho. These findings suggest that NBP treatment may be a potential strategy for ameliorating epileptogenesis and the comorbidities of cognitive and psychological impairments.

## Introduction

Epilepsy is a common chronic neurological disease with recurrent seizures and abnormal electroencephalogram (EEG) discharge, of which the annual cumulative incidence was 67.77 per 100,000 persons worldwide (Fiest et al., 2017). According to a population-based study, one-third of the people with epilepsy have associated anxiety or depressive disorder (Rai et al., 2012). In chronic epilepsies, about 70–80% of patients have cognitive impairment (Helmstaedter and Witt, 2012). Moreover, recurrent seizures or uncontrolled convulsions aggravate cognitive deficits and affect the life quality of the epilepsy patients (You et al., 2017). Furthermore, it has been reported that about 39% of patients with epilepsy are drug-resistant (Kwan and Brodie, 2000). Studies have shown that treatment with some antiepileptic drugs (AEDs) is associated with psychiatric comorbidity deterioration in the patients (Mula and Sander, 2007; Brodie et al., 2016; Mula, 2017). Therefore, attentions need to be paid in finding new effective drugs concerning epilepsy comorbidities.

L-3-*n*-butylphthalide (NBP), extracted from the seeds of *Apium graveolens* Linn. (Chinese celery), is widely applied to treat ischemic stroke (Peng and Cui, 2013). Although the specific target of NBP is unknown, accumulative studies suggest that NBP ameliorates cognitive dysfunction in ischemic animal models, as well as transgenic mouse models of Alzheimer’s disease (AD) (3xTg mice and APP/PS1 mice), by inhibiting oxidative damage, rescuing synaptic dysfunction, reducing inflammatory, and alleviating neuron loss (Peng et al., 2007, 2010; Wang et al., 2016; Zhao et al., 2016). Epilepsy and dementia/AD pathology share impairments in brain networks associated with hippocampus, and hence have similar behavioral and cognitive disturbances (Pulliainen et al., 2000; Vrinda et al., 2018). Importantly, some findings also suggest that older patients with epilepsy are at a higher risk of developing cognitive impairment and ultimately dementia (Breteler et al., 1995; Sen et al., 2018). These reports infer the therapeutic potential of NBP in the treatment of epileptic comorbidities. However, little is known about the role of NBP in epilepsy and the neurobehavioral comorbidities of epilepsy.

Given that neural synaptic proteins such as postsynaptic density protein 95 (PSD95), a scaffold protein associated with synapse maturation and synaptic stability, strength, and plasticity (El-Husseini et al., 2000; Elias et al., 2006), and glutamic acid decarboxylase 65/67 (GAD65/67), an important enzyme in gamma-aminobutyric acid (GABA) synthesis, are critical for neural synapse in cognitive dysfunction and epilepsy (Fernandez et al., 2017), and that brain-derived neurotrophic factor (BDNF) and Klotho are neuroprotective factors known to improve cognition and/or psychiatric behavior (Kang and Schuman, 1995; Sairanen et al., 2007; Dubal et al., 2014, 2015), whether these proteins were affected by NBP in the cellular processes also needs to be clarified.

In the present study, we found that NBP supplement alleviated seizure severity and abnormal EEG in pilocarpine-induced chronic epileptic mice. We also demonstrated that NBP treatment ameliorated cognitive impairment and emotional disorder in these epileptic mice. The possible mechanisms underlying the efficacy of NBP may be associated with its role in reducing neuronal loss and increase the expression of PSD95 and GAD65/67. In addition, NBP treatment increased the transcription of neuroprotective factors, BDNF and Klotho. These findings suggest that NBP may be a potential drug for ameliorating epileptogenesis and the comorbidities of cognitive and psychological impairments.

## Materials and Methods

### Drugs and Chemicals

NBP (purity >98%), offered by CSPC NBP Pharmaceutical Co., Ltd. (Shijiazhuang, China), was dissolved in 0.5% Tween-80 (Solarbio, Beijing, China) solution at a concentration of 10 mg/ml. Pilocarpine was purchased from BSZH Co., Ltd. (Beijing, China) and was dissolved in 0.9% sodium chloride.

### Animals

Fifty-seven 10-week-old male C57BL/6 mice (weighing 22–25 g) were from the Laboratory Animal Centre of Xiamen University. Animals were housed under a 12/12-h light/dark cycle (lights on at 6:00 a.m.) with food and water *ad libitum*. The temperature and humidity of the breeding house was kept consistent (temperature: 23 ± 1°C; humidity: 50–60%) during the experiments. All efforts were aimed to lessen animal’s suffering. All animal experiments were performed in accordance with the protocols of the Institutional Animal Care and Use Committee at Xiamen University.

### Pilocarpine Model of Temporal Lobe Epilepsy in Mice and Treatment

**FIGURE 1:**
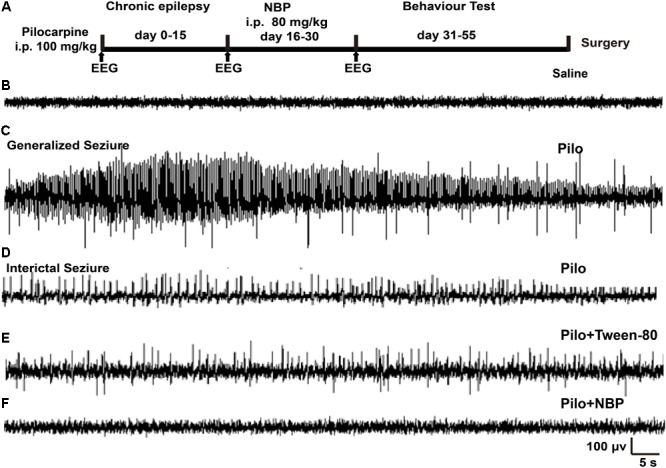
NBP treatment reduced seizure incidence. **(A)** Schematic model of the experimental design for NBP treatment using pilocarpine-induced chronically epileptic mice. **(B,C)** Examples of EEG recordings in C57BL/6 mice at 1 h following saline **(B)** or pilocarpine **(C)** treatments. Note that pilocarpine generated high-frequency spikes. **(D)** In the epileptic animals 15 days post-SE, interictal EEG during a spontaneous behavioral seizure exhibited epileptiform discharges. **(E,F)** Fifteen days after SE, mice were treated with NBP or Tween-80 for 14 days. The EEGs on the 15th day post Tween-80 or NBP injection were recorded. The spontaneous epileptiform discharges and behavioral seizures were significantly decreased in NBP treated mice **(F)** whereas they were still exhibited in the Tween-80 treated mice **(E)**.

According to the method in pilocarpine-induced status epilepticus (SE), mice were intraperitoneally (i.p.) injected with small-dose of pilocarpine (100 mg/kg, *n* = 45) or saline (*n* = 12) every 20 min until the onset of SE (Groticke et al., 2007). For blocking peripheral cholinergic effects, atropine sulfate (1 mg/kg i.p.) was administered 30 min before pilocarpine injection (Clifford et al., 1987; Morrisett et al., 1987). Epileptic behavior of the mice was observed as previously described (Racine, 1972) with the following stages: Stage I, Mouth and facial movements; Stage II, Head nodding; Stage III, Forelimb clonus; Stage IV, Rearing; Stage V, Rearing and falling. SE was referred to as a Stage IV–V motor seizure sustaining more than 30 min. All mice developed SE and were injected with diazepam (10 mg/kg) after 120 min of SE to decrease mortality. About 20/45 (44%) mice died during or after SE. Fifteen days following SE, the survival mice were randomly divided into two groups: pilocarpine + NBP group and pilocarpine + Tween-80 group. For pilocarpine + NBP group (*n* = 13), mice were administrated NBP by i.p. for 14 consecutive days at a dose of 80 mg/kg according to the reported literatures with minor modification (Peng et al., 2010; Wang et al., 2014). For pilocarpine + Tween-80 group (*n* = 12), mice received 0.5% Tween-80 i.p. injection without NBP. The 12 male mice with saline control treatment received another 0.5% Tween-80 i.p. injection for vehicle group (*n* = 12 saline + Tween-80 group). EEGs were recorded at 1 h, 15 days, and 30 days following saline or pilocarpine injection (**Figure 1A**). Eight mice of each group were then subjected to cognitive and psychological behavior test following the final injection of NBP or 0.5% Tween-80. Another four or five mice were sacrificed and the brains were dissected for Nissl staining, Western blotting or quantitative polymerase chain reaction (qPCR).

### EEGs Recording

Electroencephalograms were recorded according to the method depicted previously (Zheng et al., 2016). Briefly, two polyamide-insulated stainless steel monopolar microelectrodes (0.1 mm diameter; Plastics One, Inc., Roanoke, VA, United States) were imbedded into the frontal area of bilateral hemispheres for 1 cm of depth. Insulated 50 μm-diameter stainless steel wire (California Fine Wire) was implanted into the skin above nasal bone. The reference electrode was placed in the right musculi temporalis. Data were analyzed by Easy EEG II (Version 2.0.1).

### Behavior Tests

Behavior tests were performed between 8:00 a.m. and 2:00 p.m., i.e., during the light period of the light–dark cycle on the 31st day after SE. All data were recorded and analyzed by SMART digital tracking system (Version 3.0).

#### Morris Water Maze

Morris water maze (MWM) was performed to evaluate the memory and learning capacity (Morris, 1984). The four quadrants pool of MWM, was 90 cm in diameter and 35 cm in height. A hidden platform was placed in the target quadrant 1.0–2.0 cm under the surface of the water (22–25°C). The MWM test was carried out within 48 h after last injection and the test was composed of spatial memory training and probe trial. In the spatial memory training, all mice were trained for four times per day for 7 days. Mice were put into the water facing the wall of the pool at four different directions in the four quadrants and were allowed to find the hidden platform within 1 min standing for 10 s. If the mouse failed to find the platform within 1 min, the mouse was then guided into the platform standing for 10 s. The time for finding the platform was recorded (escape latency). The probe trial was performed 24 h after the last training session. The platform was removed and the mice were released in the zone opposite to the area of the platform, allowing them to swim freely for 60 s. The mean speed in the zone and the percentage of time in each quadrant were recorded. After testing, all mice were dried with towel and were placed in a warming cage.

#### Black–White Box

The black–white box was carried out to assess anxiety-like behavior for mice’s natural preference for dark spaces and exploring new environment spontaneously (Teixeira et al., 2011). Black–white box was made of wood, 40 cm long × 15 cm wide × 15 cm high, divided into two compartments (light and dark, accounting for 50% respectively) and connected by a small door by which the mice can pass. Each tested mouse was placed in the small door facing dark box and its behavior was recorded for a 10-min trial. The time the mice spent in the light one was analyzed to evaluate anxiety behavior.

#### Tail Suspension Test

Mice were suspended from an iron hoop by fastening a quarter of the tail with adhesive tape. The time of the animal’s immobility was recorded during the final 6 min of the 7-min test. Immobility was defined as the deficiency of any limb or body movements, beside those caused by respiration.

#### Western Blotting

All brain tissues were homogenized in RIPA buffer (Boster, Wuhan, China), with protease inhibitors and phosphatase inhibitors (Roche, Basel, Switzerland). The samples were centrifuged at 12,000 rpm for 15 min at 4°C and tissue debris was removed. Protein concentration was determined by BCA assay kit (Thermo Fisher Scientific, Waltham, United States). Protein samples (20∼40 μg per lane) were separated by SDS-PAGE and were then transferred into PVDF membranes (Millipore, Billerica, United States). Blocked for 60 min with 5% (w/v) non-fat milk in Tris-buffered solution-Tween 20, membranes were then incubated overnight at 4°C with PSD95 (1:500, Millipore, St. Charles, MO, United States), GAD65/67 (1:1000, Millipore, St. Charles, MO, United States), or β-actin (1:4000, Cell Signaling Technology, Boston, United States) primary antibody. Then they were incubated with HRP conjugated secondary anti-rabbit or anti-mouse IgG (Thermo Fisher Scientific, Waltham, United States) for 60 min at room temperature. The signals were detected using ECL kit (Millipore, St. Charles, MO, United States) and analyzed by Image J 1.46.

#### Quantitative Polymerase Chain Reaction

Total RNA was extracted using Trizol Reagent (Thermo Fisher Scientific, Waltham, United States), and was reverse transcripted to cDNA using ReverTra Ace qPCR RT Master Mix (TOYOBO, Osaka, Japan). Quantitative real-time PCR was performed using a Light Cycler 480 II (Roche, Basel, Switzerland) with SYBR Green PCR Master Mix (Roche, Basel, Switzerland) in a 10-μl reaction mixture with 250 nM primers. Relative mRNA level of examined gene was estimated using the comparative Ct method. The real-time value for each sample was averaged and compared using the CT method, where the amount of target RNA (2^-ΔΔCT^) was normalized to the endogenous *β-actin* reference (ΔCT) and then normalized against those levels in vehicle group. The primer sequences were as follows:

*Bdnf*: Forward TCATACTTCGGTTGCATGAAGG
  Reverse AGACCTCTCGAACCTGCCC
*Klotho*: Forward ACTACGTTCAAGTGGACACTACT
  Reverse GATGGCAGAGAAATCAACACAGT
*β-actin*: Forward AGTGTGACGTTGACATCCGT
  Reverse GCCAGAGCAGTAATCTCCTTC

#### Tissue Processing and Nissl Staining

Mice (*n* = 4 or 5 per group) were anesthetized and the brain was acquired, fixed in 4% paraformaldehyde for 24 h and then dehydrated consecutively with 20 and 30% sucrose at 4°C for 2 days. Brain tissues were then imbedded with OCT (Sakura, Culver City, CA, United States) at -80°C and were sectioned using a freezing microtome (Leica CM1950, Nussloch, Germany) to generate 30 μm sections. Brain sections were washed with phosphate buffer solution and were then incubated with Nissl Staining Solution (Beyotime, Shanghai, China) for 10 min. All sections were cover-slipped with mounting solution (Cwbio, Beijing, China) and inspected with a light microscope (Carl Zeiss, Göttingen, Germany). Photographs were taken with a Moticam HRC digital camera (Motic, Hong Kong, China) and the average cell number in the areas of hippocampus from five sections per mouse was quantified and analyzed by Image J 1.46.

### Statistical Analysis

Data were expressed as mean ± standard error of mean (SEM). Statistical significance was determined by one-way ANOVA and Bonferroni’s test (GraphPad Prism 5.0). *p* < 0.05 was considered significant.

## Results

### NBP Treatment Reduced Seizure Incidence

To determine the potential role of NBP in epilepsy, we first developed a chronically epileptic mouse model by i.p. injection of pilocarpine. An overview of the experiment design, which showed the timing of pilocarpine and NBP administration, was illustrated in **Figure 1A**. EEG recording was carried out at 1 h after treatment with saline control (**Figure 1B**) or pilocarpine (**Figure 1C**). In accordance with previous observation, sporadic pathological discharges and SE were observed in pilocarpine treated mice but not in the vehicle mice (saline + Tween-80 group). Fifteen days later, all the spontaneously epileptic mice confirmed by interictal seizure EEG (**Figure 1D**) were then divided into two groups and were injected with NBP or Tween-80 for another 14 days. EEG recordings on the 30th day showed that NBP significantly ameliorated the epileptiform activity in epileptic mice (pilocarpine + NBP group; **Figure 1F**) whereas there was no change in the pathological discharges in the Tween-80-treated mice (pilocarpine + Tween-80 group; **Figure 1E**), suggesting a potential protective role of NBP in epileptogenesis.

### NBP Improved Spatial Learning and Memory Deficits in Pilocarpine-Induced Epileptic Mice

**FIGURE 2:**
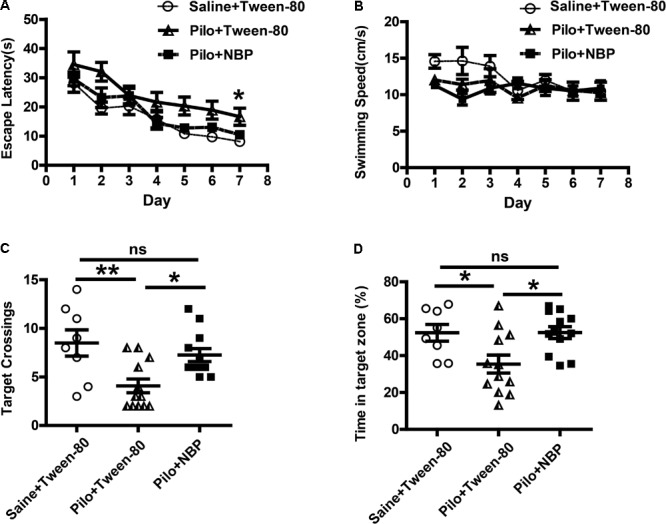
NBP treatment improved spatial learning and reverted memory deficits in chronically epileptic C57BL/6 mice. **(A–D)** Morris water maze test. **(A)** Mean latency to reach platform with a 7-day training period. **(B)** Swimming speed. **(C)** Times of target platform crossing. **(D)** The percentage of time in target zone. *n* = 8 for each group. ^∗^*p* < 0.05, ^∗∗^*p* < 0.01, ^∗∗∗^ *p* < 0.001; ns, not significant.

Epilepsy is complicated by neurobehavioral comorbidities, including cognitive impairment, psychiatric disorders, and social problems (Lin et al., 2012). Therefore, we examined whether NBP could improve behavioral phenotypes associated with the pilocarpine model. We first investigated the effects of NBP on mouse performance in MWM test, which requires the use of external visual cues to locate a hidden platform and to escape the water. The mean speed in the three groups animals showed no preference for each other, so that the possibility that speed might influence water maze performance in these animals can be excluded (**Figure 2B**). During a hidden platform test (acquisition), epileptic animals that treated with NBP showed significantly shorter escape latencies compared to pilocarpine treated epileptic mice (**Figure 2A**), similar to that in control group. This improvement was confirmed in a probe test 24 h following the final testing session (**Figures 2C,D**). The test showed that NBP treated mice made numerous platform crossings and spent significantly more time in the target quadrant (**Figures 2C,D**). NBP application rescued these spatial learning and memory deficits in pilocarpine-induced epileptic mice.

**FIGURE 3:**
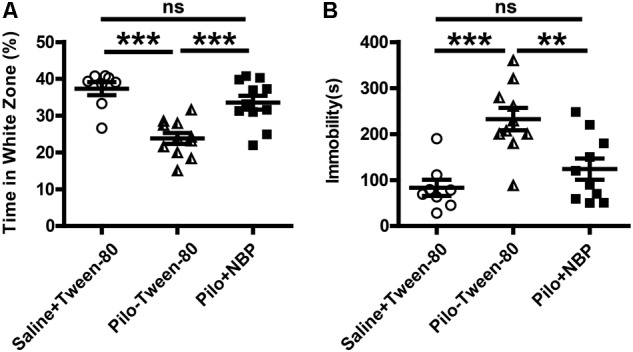
NBP treatment ameliorated anxiety- and depression-like behavior in pilocarpine induced epileptic mice. **(A)** Black–white box: time spent in white compartment. **(B)** Total time of immobility in the tail suspension test during the last 6 min of the 7 min test session. *n* = 8 for each group. ^∗∗^*p* < 0.01, ^∗∗∗^*p* < 0.001; ns, not significant.

Anxiety- and depression-like behaviors in animals were assessed by the black–white box test or tail suspension test. During a 10-min test (locomotion), mice in vehicle group (saline + Tween-80 group) or pilocarpine + NBP group displayed hyperactivity and spent more time in the white box than those in pilocarpine + Tween-80 group (**Figure 3A**). The tail suspension test showed that NBP treated mice (pilocarpine + NBP group) had a profound increase in their reaction to suspension, similar to those in vehicle group, whereas the pilocarpine treated epilepsy mice (pilocarpine + NBP group) developed a characteristic immobile posture following the platform withdrawal (**Figure 3B**).

### NBP Up-Regulated the Expression of PSD95 and GAD65/67 in Epileptic Mice

**FIGURE 4:**
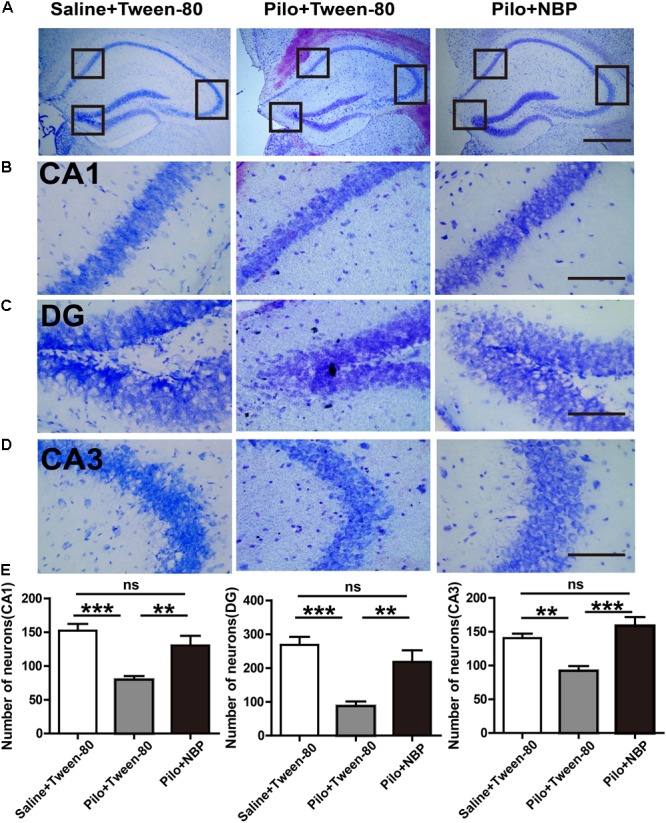
NBP treatment decreased the hippocampal neuronal loss in chronically epileptic mice. **(A)** Representative Nissl staining in the hippocampus of the mouse brain. Scale bar = 50 μm. **(B–D)** Representative images of the Nissl staining in the DG, CA3, and CA1 areas with maximum projection were shown. **(E)** Average cell number in the areas of hippocampus from five sections per mouse was quantified. Scale bar = 10 μm. *n* = 4 or 5 for each group. ^∗^ *p* < 0.05, ^∗∗^*p* < 0.01, ^∗∗∗^*p* < 0.001; ns, not significant.

**FIGURE 5:**
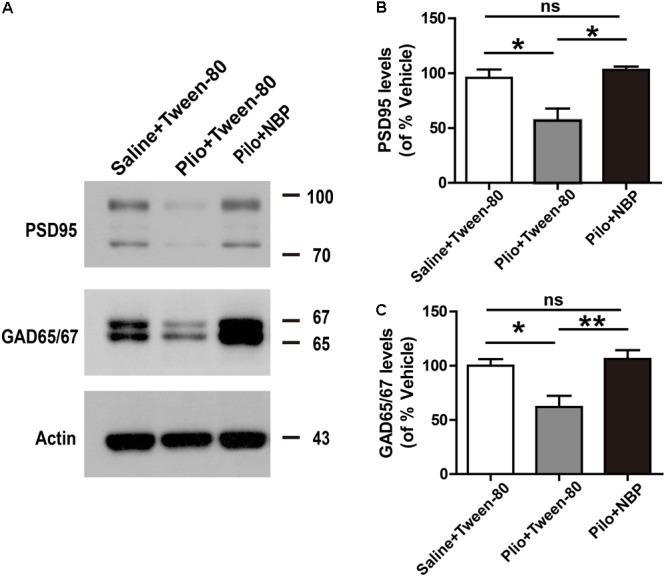
Administration of NBP up-regulated the expression of PSD95 and GAD65. **(A)** Representative Western blotting images of brain extracts; **(B,C)** NBP treatment increased the protein level of PSD95 and GAD65 in epileptic mouse brain. *n* = 4 or 5 for each group. ^∗^*p* < 0.05, ^∗∗^*p* < 0.01; ns, not significant.

We next asked whether NBP affected neuronal loss in the hippocampus of epileptic mice. Nissl staining showed that Nissl positive neuron number in the hippocampal DG, CA3, or CA1 areas significantly decreased, and were restored in response to NBP treatment in epileptic mice (**Figures 4A–E**), suggesting that the dramatic protective effect of NBP treatment was due to a reversal of neuronal loss in the hippocampus. PSD95, a scaffold protein, is associated with synapse maturation and synaptic stability, strength, and plasticity (El-Husseini et al., 2000; Elias et al., 2006). Additionally, PSD95 expression is reduced in cognitive dysfunction and epilepsy (Zhang et al., 2014; Fernandez et al., 2017). GAD65/67, an important enzyme in GABA synthesis, is related to neurologic disorders, such as epilepsy, ataxia, cognitive impairment and emotion disorder (Dayalu and Teener, 2012; Muller et al., 2015; Qi et al., 2018). To clarify the cellular and synaptic mechanisms underlying the divergent effects of NBP application *in vivo*, the expression levels of PSD95 and GAD65/67 in animals were evaluated by Western blotting. We detected significantly decreased expression levels of PSD95 and GAD65/67 in the brain of mice treated with pilocarpine, and significantly increased levels of them in NBP treated mice (pilocarpine + Tween-80) (**Figure 5**).

**FIGURE 6:**
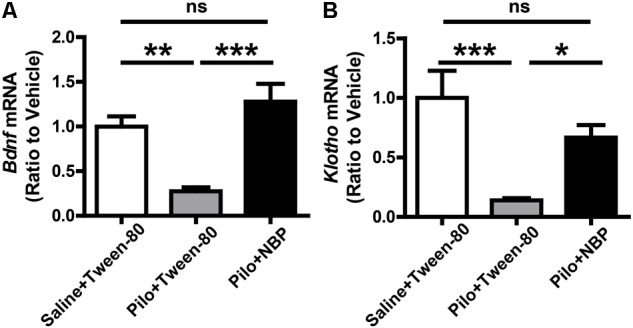
NBP treatment enhanced *Bdnf* and *Klotho* mRNA level. **(A,B)** NBP up-regulated the mRNA levels of *Bdnf* **(A)** and *Klotho* **(B)** in epileptic mouse brain as quantified by qRT-PCR. *n* = 4 or 5 for each group. ^∗^*p* < 0.05, ^∗∗^*p* < 0.01, ^∗∗∗^*p* < 0.001; ns, not significant.

Furthermore, BDNF is an important neurotrophic factor that enhances synapse formation and cognitive functions (Parkhurst et al., 2013; Karpova, 2014), and *Klotho* is already known to improve cognition (Dubal et al., 2014, 2015). When compared to epileptic mice without NBP treatment (pilocarpine + Tween-80), the mRNA levels of *Bdnf* and *Klotho* were strongly up-regulated in NBP treated epileptic mice (**Figure 6**) whereas NBP alone did not affect *Bdnf* expression (Supplementary Figure 1). These results provide direct biochemical evidence that systemic treatment with NBP can mitigate the epileptiform activity in epileptic mice.

## Discussion

Recurrent seizures lead to severe anxiety or depression, which is a major reason for cognitive decline (Lagae, 2006; Kimiskidis et al., 2007; Gaitatzis et al., 2012; de Toffol et al., 2017). Preventing seizures or mitigating symptoms of anxiety or depression are clinical need. Several studies have reported the protective role of NBP in central nervous system (Peng et al., 2010; Zhang et al., 2016), but few researches have discussed the effect of NBP on epilepsy. In this study, pilocarpine-induced chronic epilepsy model was used to investigate the effects of NBP on epilepsy associated anxiety, depression, and cognitive deficit (Inostroza et al., 2011; Pineda et al., 2014). Our findings suggest that NBP treatment reduced spontaneous spike-waves in chronic epilepsy. Furthermore, our results show that NBP plays a vital role in antidepressant, antianxiety and ameliorating learning and memory impairment by rescuing the chronic epilepsy-induced neuronal loss in CA1, CA3, and DG areas of hippocampus.

Here, the EEG recording showed that the occurrence of spontaneous discharge decreased in epileptic mice in response to NBP treatment. This is consistent with previous study that NBP could keep excitatory and inhibitory neuronal systems in balance in acute epileptic mice brain (Yu et al., 1988; Han et al., 2016). Additionally, imbalance between excitatory and inhibitory neurotransmitters, such as glutamic acid (Glu) and GABA, results in epileptogenesis (Svenningsen et al., 2006; Hao et al., 2016). GAD65/67, a GABA-synthesizing enzyme, decides GABA levels in postnatal synapse maturation (Horovitz et al., 2012; Fouka et al., 2015). Thus, our results revealed that the antiepileptic effect of NBP might be resulted from the up-regulation of GAD65/67 and the reversion of neuronal loss.

It has also been reported that NBP promotes neurogenesis and is neuroprotective against neuronal apoptosis, and improves synaptic plasticity (Chang and Wang, 2003; Yang et al., 2015). Furthermore, studies have shown that NBP improved cognitive deficit in a transgenic model of AD (Peng et al., 2010). What’s more, recent studies indicate that AD and epilepsy had similar mechanism in the pathogenesis of cognitive impairment (Corbett et al., 2017; You et al., 2017). These results suggest that NBP might serve as a potential therapeutic drug in the treatment of epileptic comorbidities. Here, we found that NBP treatment enhanced the learning and memory capacity in chronic pilocarpine-induced model by MWM test. In addition, we observed an up-regulated level of synapse-associated protein in response to NBP treatment. PSD95 plays an important role in synapse stabilization and plasticity (El-Husseini et al., 2000). The decrease in PSD95 levels are highly correlated with learning and memory impairments (Chen et al., 1998; Migaud et al., 1998), and PSD95 is down regulated in epileptic activity (Wyneken et al., 2001). Corroborating these researches, the up-regulation of PSD95 in response to NBP treatment in epileptic mice may contribute to the improvement of cognitive function.

In this study, we also found that NBP may have therapeutic effects on anxiety and depressive behavior by increasing *Bdnf* and *Klotho* mRNA level in pilocarpine-induced epileptic mice. There is now ample evidence that BDNF, a neurotrophic factor important in promoting immature neurons development, increasing the survival of adult neurons and synaptic plasticity (Kang and Schuman, 1995; Sairanen et al., 2007), is protective in antidepressant and antianxiety. Treatment with antidepressant or antianxiety drugs can restore *Bdnf* mRNA level in stress-induced model (Smith et al., 1995; Kozisek et al., 2008). Although it has been reported that BDNF expression was enhanced in pilocarpine-induced SE (Thomas et al., 2016), BDNF has also been found decreased in patients with chronic temporal lobe epilepsy (TLE) and in animal model of chronic cyclothiazide seizure (Kong and Cheng, 2014; Chen et al., 2016). Besides, Xiang et al. (2014) pointed out that NBP alleviated cognitive dysfunction in APP mice by BDNF/TrkB/PI3K/AKT pathway. Moreover, it has been reported that *Klotho* is reduced in TLE and its downregulation is involved in neurodegenerative disorders and inflammation (de Oliveira, 2006; Abraham et al., 2012). Consequently, we speculate the therapeutic function of NBP in chronic epileptic comorbidities associated with anxiety and depressive behavior by increasing the expression level of BDNF and Klotho.

Collectively, this study indicates that treatment with NBP could be a potential strategy to slow down or even to reverse chronic epilepsy and the epileptic comorbidities such as cognitive decline and psychological impairments. However, the molecular mechanisms for the application of NBP in epilepsy still need to be further investigated. In addition, the role of NBP in acute epilepsy was unclear. And there are also limitations for the use of atropine sulfate to block peripheral cholinergic effects in this study as it can cross the blood–brain barrier and in this way it might affect brain functions. NBP has already been approved by the State Food and Drug Administration (SFDA) of China for clinical use in stroke patients since 2002. This study suggests that therapeutic strategies of NBP for chronic epilepsy and the comorbidities may expand the applicative scope of this drug. However, additional experimental data are needed to prove the antiepileptic effects of NBP, as well as the dose-dependent responses of NBP in epilepsy.

## Author Contributions

WZ and HZ conceived and designed the study. XY, ZR, and YC performed the experiments and analyzed the data. XY, ZR, and HZ wrote the paper. HL, XW, XH, and BC coordinated the study and provided technical assistance. ZL, YL, and Y-wZ revised the paper. All authors reviewed the results and approved the final version of the manuscript.

## Conflict of Interest Statement

The authors declare that the research was conducted in the absence of any commercial or financial relationships that could be construed as a potential conflict of interest.
