# Supplementary material for: Protective Role of L-3-n-Butylphthalide in Cognitive Function and Dysthymic Disorders in Mouse With Chronic Epilepsy
Source: Front Pharmacol. 2018 Jul 11;9:734. doi: 10.3389/fphar.2018.00734 (PMC6051017; doi:10.3389/fphar.2018.00734)
Supplement: Supplementary file 1 [file Data_Sheet_1.PDF]

Supplementary Material

Protective role of L-3-n-butylphthalide in cognitive function and dysthymic disorders in mouse with chronic epilepsy

Xiaowen Ye[1,2*], Zhouyi Rong[2*], Yanfang Li[2*], Xintian Wang[1,2,3], Baoying Cheng[2], Yiyun Cheng[1,2,3], Haijuan Luo[1,2], Yue Ti[4], Xiaohua Huang[5], Zhaoji Liu[1,2], Yun-wu Zhang[2], Weihong Zheng[1,3,4‡], Honghua Zheng[2,5,6‡]

[‡]**Correspondence:** Honghua Zheng, Phone/Fax: (86) 0592-2880582; E-mail: honghua@xmu.edu.cn; or Weihong Zheng, Phone/Fax: (86) 0592-2590120; E-mail: zwh610547@xmu.edu.cn.

1 Supplementary Figure 1

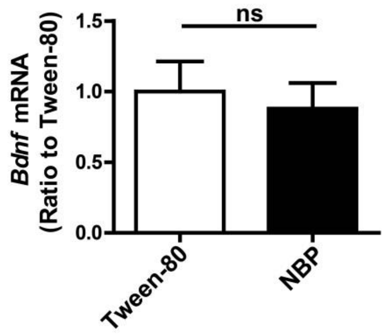

Ten-week-old male C57BL/6 mice (n=8) were randomly intraperitoneally injected with NBP (80mg/kg) or Tween-80 for 7 days. Effect of NBP on the expression of *Bdnf* in C57BL/6 mouse brain was detected. The relative *Bdnf* mRNA level in NBP treated group was comparable to that of Tween-80 treated group. n=4 for each group, ns: not significant, *Student's t test.*
